# Landolfi’s Sign: A Riddle for Primary Care Physicians

**DOI:** 10.4103/jopcs.jopcs_14_20

**Published:** 2020-12-31

**Authors:** Ritwik Ghosh, Swagatam Sengupta, SK Minhajuddin Siraj, Julián Benito-León

**Affiliations:** 1 Department of General Medicine, Burdwan Medical College, Burdwan, West Bengal, India; 2 Department of Gynecology, Burdwan Medical College, Burdwan, West Bengal, India; 3 Department of Cardiology, Superspeciality Wing Hospital, Anamay, Burdwan, West Bengal, India; 4 Department of Neurology, University Hospital “12 de Octubre,” Madrid, Spain; 5 Centro de Investigación Biomédica en Red Sobre Enfermedades Neurodegenerativas, Madrid, Spain; 6 Department of Medicine, Universidad Complutense, Madrid, Spain

**Keywords:** Aortic regurgitation, Landolfi’s sign

## Abstract

Landolfi’s sign, alternating systolic constriction and diastolic dilatation of pupils, is a clinical hallmark of aortic regurgitation. It is thought to stem from exacerbation of physiological circulatory hippus in the vessels of iris due to a wide pulse pressure in a backdrop of severe aortic valvular incompetence. Degenerative and rheumatic heart diseases are exquisitely common in rural India and often these patients turn up late with complications to the primary care physicians. Herein, the authors report a 34-year-old pregnant female who presented with acute heart failure, and on examination, Landolfi’s sign was found. It was immediately followed by Doppler echocardiography to stamp it as a case of severe aortic regurgitation. The patient was stabilized with anti-failure medications and feto-maternal health was closely monitored. The authors want to conclude claiming that bedside clinical training in cardiology will forever remain important, more so, while dealing patients at non-sophisticated primary health-care facilities. Besides, they also argue that basic tool supports like an echocardiography should be made available at those centers.

## Introduction

Landolfi’s sign is one of the popular eponyms used to describe a clinical sign of aortic regurgitation.^[1,2]^ It is presumably an exaggeration of physiological circulatory hippus in the iridal vessels because of higher stroke volume and wider pulse pressure that is seen in severe aortic regurgitation.^[3]^ It gives rise to the phenomenon of rapid systolic contraction and diastolic dilatation of the pupils, which is synchronous with the cardiac rhythm.^[3]^ However, medical literature has curtailed relevance of this sign in the current practice due to lack of evidence.^[4]^ Still in the era of modern technology, meticulous physical examination and demonstration of eponymous signs of aortic valve disease aid and abet our memories and remind us of our cultural and professional continuity of knowledge.

## Case Report

**Figure 1:**
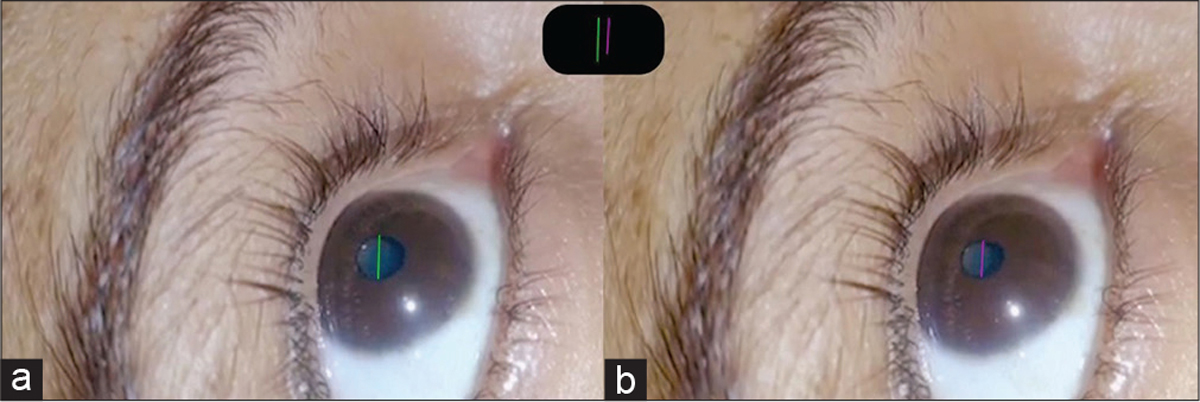
Alternating dilatation (a) and constriction (b) of the pupils occurring in synchrony with cardiac rhythm (diastole and systole, respectively)

**Figure 2:**
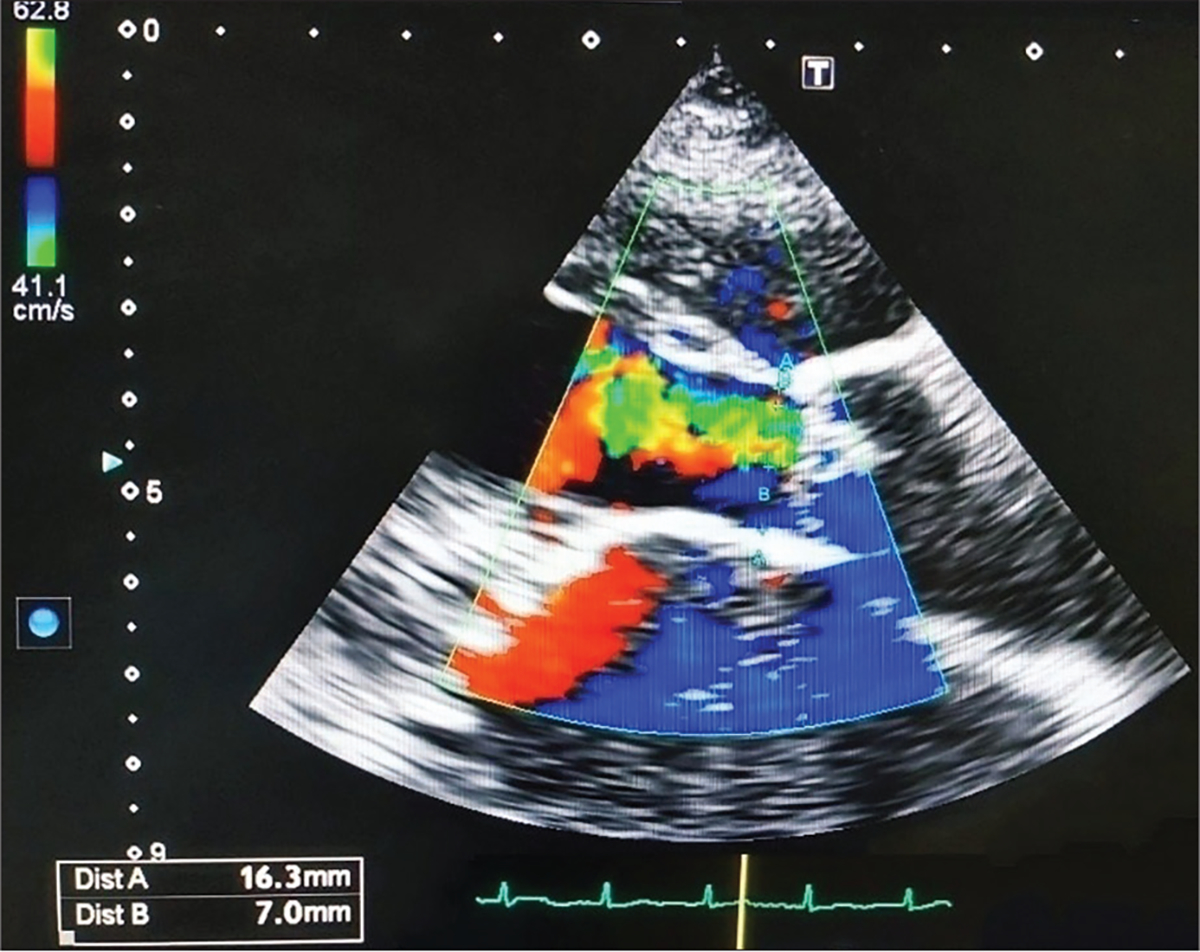
Parasternal long axis view using color Doppler flow imaging of aortic valve showing severe aortic regurgitation with vena contract width of 7 mm

A 34-year-old primigravida at 30 weeks of gestation, from rural India belonging to lower socio-economic strata was referred from a block primary health center to the obstetric emergency of our institute with progressively worsening dyspnea (New York Heart Association class 3), orthopnea, and extreme fatigability. It was associated with intermittent palpitations and two episodes of pre-syncope. On physical examination, she had features of acute heart failure (hypoxemia, pitting pedal and parietal edema, raised jugular venous pressure, tender hepatomegaly, bi-basal rales, and decreased renal output). A grade grade 3/6, soft, high-pitched, early diastolic, blowing, decrescendo murmur was heard best at the third intercostal space on the left (Erb’s point) at the end of expiration with the patient sitting up and leaning forward. Pulse pressure was wide with bounding femoral and carotid arterial pulses. Hill’s sign was positive. Careful examination of her eyes revealed alternating dilatation [Figure 1a] and constriction [Figure 1b] of the pupils occurring in synchrony with cardiac rhythm (diastole and systole, respectively). It was confirmed to be Landolfi’s sign [Video 1 and Figure 1]. Two dimensional and Doppler echocardiography revealed the presence of severe aortic regurgitation along with other cardinal features of rheumatic heart disease [Figure 2]. The patient was treated in the cardiac intensive care unit with anti-failure medications. The feto-placental profile was regularly monitored to intervene if needed to avoid fetal demise. Her hemodynamic parameters were stabilized and she was sent to higher facilities for the management of her cardiac illness with advanced obstetric care as our hospital was not equipped with these facilities.

## Discussion

Exact mechanism of hippus is yet to be elucidated.^[5]^ Recent evidence suggests that hippus is generated from complex interplay between parasympathetic and sympathetic nervous system activities at the pupil.^[5]^ The altered hemodynamic state in presence of elevated stroke volume, systolic hypertension, and low diastolic pressure give rise to wider pulse pressure resulting in exacerbation of hippus, which in this index case is considered as Landolfi’s sign. It is a rarely reported finding in the Western developed world and even in Asian subcontinent nowadays.

Modern medicine is in the era of interventional cardiology and multi-modality cardiac imaging (including nuclear cardiology).^[6]^ Still in several rural areas of India, facilities for even a simple two-dimensional echocardiography are only available at apex centers (tertiary health care centers). Hence, in these areas, rheumatic and non-rheumatic heart diseases are solely diagnosed on clinical basis by experienced primary care doctors. With the advent of evidence-based medicine, the training and expertise in clinical cardiology is gradually but certainly losing its place. Therefore, it is often seen that cases of valvular heart diseases are not being diagnosed until very late and overtly symptomatic at the primary health facilities.

## Conclusion

This case emphasizes on the fact that an observation as simple, as a pupillary exam examination, can guide the first-to-contact clinician to a life-threatening diagnosis. Our case demonstrating Landolfi’s sign is dedicated to honor the rigorous clinical training in bedside cardiology in pre- and post-echocardiography era. Besides, it also highlights the importance of availability of the basic cardiac imaging tools at primary health-care facilities.

## Supplementary Material

Video
